# Engineering plants for a changing climate

**DOI:** 10.1371/journal.pbio.3002243

**Published:** 2023-07-19

**Authors:** Joanna Clarke, Pamela C. Ronald

**Affiliations:** 1 Public Library of Science, San Francisco, California, United States of America and Cambridge, United Kingdom; 2 Department of Plant Pathology and the Genome Center, University of California, Davis, Davis, California, United States of America; 3 The Joint Bioenergy Institute, Emeryville, California, United States of America; 4 The Innovative Genomics Institute, University of California, Berkeley, Berkeley, California, United States of America

## Abstract

Climate change is affecting the types of plant varieties we can cultivate, as well as how and where we can do so. A new collection of articles explores the twin challenges of engineering plants for resilience to climate change and enhancing their carbon-capture potential.

The relationship between people and the plants we use for food, shelter, tools, medicines, clothing, and fuel dates back to before the dawn of modern humans. From the early days of gathering wild plants, through domestication, advances in cultivation, and selective breeding, to today’s bioengineering approaches, we have always had a close relationship with plants and have reaped the benefits they, and the land they grow on, can provide. The Earth’s climate is now changing at a rapid pace, affecting the domesticated crops that we rely on for food. How can we help crop plants adapt to this rapid change? And how can we use crops to help us ameliorate climate change?

Bioengineering of plants, soil, and the microbes that live among them has been put forward as one possible way to mitigate the effects of climate change. Although using biotechnology to engineer plants for resilience to a changing environment is not new [1], as the threat of climate change grows [2], so too must the attention we give to potential solutions. This issue of *PLOS Biology* therefore features a collection of articles that examine the prospect of engineering the plants we rely on to help them adapt to a changing climate. They focus on 2 major issues: creating climate-resilient varieties and increasing the carbon-capture potential of croplands.

The ongoing challenge of feeding a growing population in a socioeconomically and environmentally appropriate manner has put a spotlight on plant breeding. In this issue, Flint-Garcia and colleagues [3] highlight the potential of transferring beneficial traits (such as resistance to pests or drought tolerance) from wild relatives of crops to modern cultivars, while avoiding less desirable traits (such as small yields or tough seed coats). In a related approach, McNally and Henry discuss the success of the 3,000 Rice Genomes Project [4] and present tools that can be used to analyze the International Rice Genebank for genes that confer climate resilience [5]. But discovering the right genes and transferring these genes to or editing these genes in crops is only the beginning of the process. Being able to reach markets with engineered plants that have the desired traits requires buy-in from farmers, national breeding programs, agritech companies (discussed by Feuillet and Eversole [6]), and policy makers, as well as public support (discussed by Archibald and colleagues [7]).

One molecular target for engineering is photosynthesis, which is attractive because its enhancement holds the dual promise of increasing plant yields and capturing more atmospheric carbon [8]. In a Perspective, Matthews looks at the current state of photosynthesis engineering and discusses what could be achieved if current experimental approaches are successful [9]. Alamos and Shih explore the idea of engineering plants for increased carbon capture by examining the role of synthetic biology in plant engineering and how knowledge gained from efforts to engineer photosynthesis can be applied to the challenge of engineering root systems [10]. Fierer and Walsh discuss how soil and root microbiomes could be manipulated to increase soil carbon sequestration in croplands and address the question of whether we can increase carbon persistence in the soil [11].

These different perspectives are critical for moving forward, as there is no clear community agreement on which approach will be more effective. We hope these articles will stimulate discussions and contribute to the discovery of solutions to increase plant resilience to climate change. As you conduct further research on this topic, we would like to invite you to consider *PLOS Biology* as a possible venue for publication. The tools presented here will need to be used in concert with each other and with tools yet to be developed to enable a future agriculture that is resilient in the face of a changing climate.
